# Interstitial Fluid Flow: The Mechanical Environment of Cells and Foundation of Meridians

**DOI:** 10.1155/2012/853516

**Published:** 2012-12-17

**Authors:** Wei Yao, Yabei Li, Guanghong Ding

**Affiliations:** Department of Mechanics and Engineering Science, Shanghai Research Center of Acupuncture, Fudan University, 220 Handan Road, Shanghai 200433, China

## Abstract

Using information from the deep dissection, microobservation, and measurement of acupoints in the upper and lower limbs of the human body, we developed a three-dimensional porous medium model to simulate the flow field using FLUENT software and to study the shear stress on the surface of interstitial cells (mast cells) caused by interstitial fluid flow. The numerical simulation results show the following: (i) the parallel nature of capillaries will lead to directional interstitial fluid flow, which may explain the long interstitial tissue channels or meridians observed in some experiments; (ii) when the distribution of capillaries is staggered, increases in the velocity alternate, and the velocity tends to be uniform, which is beneficial for substance exchange; (iii) interstitial fluid flow induces a shear stress, with magnitude of several Pa, on interstitial cell membranes, which will activate cells and lead to a biological response; (iv) capillary and interstitial parameters, such as capillary density, blood pressure, capillary permeability, interstitial pressure, and interstitial porosity, affect the shear stress on cell surfaces. The numerical simulation results suggest that in vivo interstitial fluid flow constitutes the mechanical environment of cells and plays a key role in guiding cell activities, which may explain the meridian phenomena and the acupuncture effects observed in experiments.

## 1. Introduction

Interstitial fluid flow is the movement of fluid through the extracellular matrix of tissues, often between blood and lymphatic vessels. This flow provides a necessary mechanism for transporting large proteins through the interstitium and constitutes an important component of microcirculation [1]. Apart from its role in mass transport, interstitial fluid flow also provides a specific mechanical environment that is important for the physiological activities of interstitial cells [2, 3]. Several in vitro experiments showed that interstitial fluid flow was very important for cell activities and that a flow of *μ*m/s magnitude induced physiological responses from cells [4–8]. In vitro numerical simulations of how the architecture of extracellular fibers affects the shear stress on cell membranes also showed that interstitial fluid flow is important to the fluid force on a cell imbedded in a 3D matrix [9, 10]. Blood flow plays an important role in guiding the physiological activities of endothelial cells (ECs) and smooth muscle cells (SMCs) and during bone remodeling [11–13]. However, studies of the effect of interstitial fluid flow on interstitial cells (mast cells) are rare. Mast cells are a type of immune cell found in connective tissues. When mast cells are stimulated, they release chemical mediators from their cellular granules into the extracellular matrix and initiate a series of biological responses; many of the responses are correlated with acupuncture effects [14]. 

Until now, there were no direct in vivo measurements of interstitial fluid flow, and information regarding interstitial fluid flow has only been inferred from other possibly correlated measurements. For example, Li et al. visualized regional hypodermic migration channels in the interstitial fluid of humans using magnetic resonance imaging (MRI) [15]. These channels were different from those of lymphatic or blood vessels and partially coincided with the characteristics of meridians. It is believed that interstitial fluid flow can be used to help illustrate the modern physiological mechanism of meridians. In another experiment, nuclide was injected into the “Taiyuan” acupoint of a recently deceased monkey (within a half hour of death). No transmission track was found along the meridian, but a track appeared when saline solution with heparin was infused simultaneously through an axillary artery and a vein [16]. This phenomenon demonstrates that the movement of an isotope along meridians requires an impetus, which is provided by circulating blood in living beings. We proposed a dynamic model to simulate the interstitial fluid flow near meridians and discovered that the source of direct impetus for this flow was the penetration of plasma between capillaries and interstitial fluid [17]. Furthermore, we developed a two-dimensional (2D) model to study the flow field in connective tissues and observed directional fluid flow [18, 19].

MRI revealed that the tip of an acupuncture needle is normally placed near an interosseous membrane. In this paper, we will investigate the three-dimensional (3D) flow pattern of interstitial fluid in a human interosseous membrane, the effect of physiological parameters on flow velocity and the physiological impact of flow on interstitial cells. The rest of the paper is organized as follows. In Section 2, we present a 3D porous media model to describe the interosseous membrane. The results obtained from the proposed model are presented in Section 3. A discussion and summary of the present study are provided in Section 4.

## 2. Model and Methods

### 2.1. The 3D Model

**Figure 1:**
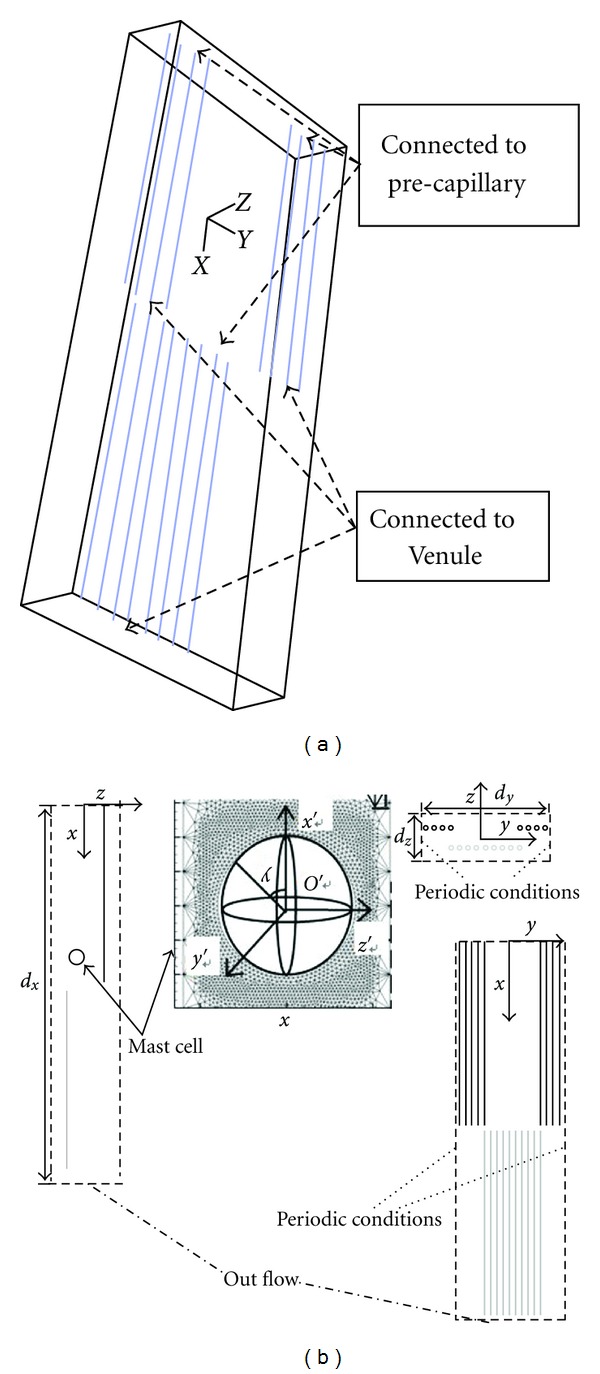
Model of the interosseous membrane. (a) The sketch map; (b) 3D porous media model.

The interosseous membrane is 25 cm long, 2 cm wide, and 0.2 cm thick. Anatomical observations showed that capillaries formed clusters and were distributed in alternating layers. In each cluster, the capillaries were nearly parallel.  Figure 1(a) is the sketch map of one membrane unit. The upstream ends of the capillaries connected to a pre-capillary, and the downstream ends connected to a venule. The average length of a capillary group is 2000 *μ*m (see the *x*-axis in Figure 1(a)), and the average width is 400 *μ*m (see *y*-axis in Figure 1(a)). The distance between nearby clusters is approximately 1875 *μ*m along the *x*-axis and approximately 315 *μ*m along the *y*-axis. Suppose the distance between the capillaries in a cluster is equal, which is approximately 50 *μ*m. According to these characteristics, we develop a 3D model, shown in Figure 1(b). The solid thick lines represent capillaries, and the black and grey colors indicate different layers. The dashed rectangle marks the calculation domain.

To study the effect of flow on interstitial cells, we place a mast cell (a sphere with a diameter of 16 *μ*m) in the center of the domain (the center of the sphere is at *x* = 2 mm, *y* = *z* = 0 mm) (Figure 1(b)). The magnified mast cell in Figure 1(b) shows the local Cartesian coordinates *O*′*x*′*y*′*z*′. The origin (*O*′) is at the center of the sphere, and the *x*′-axis, *y*′-axis and *z*′-axis are parallel to the *x*-axis, *y*-axis and *z*-axis, respectively. For mast cells in the interstitium, there is a thin boundary layer near the cell surface, called the Brinkman boundary layer. Therefore, during mesh generation, there is a refined zone near the surface of the mast cell, shown in Figure 1(b) [20].

### 2.2. Governing Equations

 Because the Reynolds number of the interstitial fluid flow is small, inertia can be neglected. The space in the interstitium composed of parallel collagen fibrils is assumed to be a porous medium. The governing equations are the Brinkman and continuity equations [20]:

(1) $$\begin{array}{c} \nabla p=\mu \Delta \overset{⃑}{u}-\frac{\mu }{k_{p}}\overset{⃑}{u}, \end{array}$$

(2) $$\begin{array}{c} \nabla \cdot \overset{⃑}{u}=\overset{⃑}{0}, \end{array}$$

where ∇ is the gradient operator, Δ is the Laplacian operator, *u* is the local flow velocity vector, *p* is the interstitial pressure, *μ* is the viscosity of interstitial fluid, and *k*_*p*_ is the Darcy permeability of the collagen fibril matrix. The term on the left-hand side of (1) is the pressure gradient. The first term on the right-hand side represents the viscous term, and the last term on the right-hand side represents the Darcy-Forchheimer term, which characterizes flow in a porous medium.

The dimensionless variables are defined as ${\overset{⃑}{u}}^{*}=\overset{⃑}{u}/U$; p* = *p*/*ρU* ^2^; ${\overset{⃑}{x}}^{*}=\overset{⃑}{x}/D$; and $\overset{⃑}{k}={\overset{⃑}{k}}_{p}/D^{2}$, where *U* is the characteristic velocity, *D* is the diameter of capillaries, *ρ* is the fluid density, and $\overset{⃑}{x}$ is the position vector. Equations (1) and (2) are rewritten in dimensionless forms as:

(3) ∇p∗=1ReΔu⃑∗−1Re·ku⃑∗,

(4) $$\begin{array}{c} \nabla \cdot {\overset{⃑}{u}}^{*}=\overset{⃑}{0}, \end{array}$$

where Re is the Reynolds number, defined as *Re* = *ρU* *D*/*μ*. In our model, ${\overset{⃑}{k}}_{p}$ is small (10^−16 ^m^2^), and the dimensionless parameter $\overset{⃑}{k}$ is approximately 10^−6^. Therefore, the viscous term is small compared with the Darcy-Forchheimer term and can be neglected. Equation (3) reduces to Darcy's law [20]:

(5) ∇p∗=−1Re·ku⃑∗.

For the mast cell in the interstitium, the next results will show that the flow field near the cell is almost symmetrical about the *x*′-axis. Therefore, we use spherical coordinates to analyze the shear stress on the mast cell (*τ*_cell_), and the dominant stress is

(6) $$\begin{array}{c} \tau_{r\theta }=-\mu {\left\{r\frac{\partial }{\partial r}\left(\frac{u_{\theta }}{r}\right)+\frac{1}{r}\frac{\partial u_{r}}{\partial \theta }\right\}}_{\text{cell}}. \end{array}$$

### 2.3. Effect of Capillaries on the Interstitial Fluid Flow

Starling's hypothesis that fluid movement across microvascular walls is determined by the transmural difference in hydrostatic and oncotic pressures has become a general principle of physiology [21]:

(7) $$\begin{array}{c} v=k_{c}\left[\left(p_{c}-p_{i}\right)-\left(\pi_{c}-\pi_{i}\right)\right], \end{array}$$

where *k*_*c*_ is the permeability coefficient of the capillary walls, *p*_*c*_ is the hydrostatic pressure in blood, *p*_*i*_ is the interstitial hydrostatic pressure at the capillary wall, *π*_*c*_ is the osmotic pressure in blood, and *π*_*i*_ is the interstitial osmotic pressure at the capillary wall. Hu and Weinbaum determined that *π*_*i*_ and *p*_*i*_ (hydrostatic and colloid osmotic pressures, respectively, behind the surface glycocalyx in their model) can differ greatly from the corresponding pressures in the interstitial space [25]. Generally, it is assumed that only *p*_*c*_ varies and that *p*_*c*_ decreases linearly from the pre-capillary side to the venule side along the length of the capillary. Other parameters (*p*_*i*_, *π*_*c*_ and *π*_*i*_) are assumed to be constant [21]. Defining the characteristic velocity *U* = *k*_*c*_[(*p*_*a*_ − *p*_*i*_) − (*π*_*c*_ − *π*_*i*_)], where *p*_*a*_ is the intravascular capillary pressure at the upstream end, and using the values of the parameters listed in Table 1, the transmural capillary velocity is

(8) $$\begin{array}{c} v=U\left(1-1.5\frac{x}{L}\right), \end{array}$$

where *x* is the distance from the upstream end, and *L* is the length of the capillary.

### 2.4. Effect of Collagen Fibrils on the Interstitial Fluid Flow

Parallel collagen fibrils can influence the interstitial fluid flow. Chen et al. developed 2D and 3D finite element models analogous to the parallel collagen fibril arrays in ligaments and tendons to simulate transverse and longitudinal interstitial fluid flows [22]. The flow along collagen fibrils is defined as longitudinal flow with the flow perpendicular to collagen fibrils defined as transverse flow. The computational results provided empirical expressions for Darcy's permeability as a function of the porosity *ϕ*. Considering the fluid to be a Newtonian fluid, the empirical expressions are shown as follows:

(9) transverse  permeability:ky=kz  =1.2×10−15ϕ0.5(ϕ−ϕmin⁡)2.5,longitudinal  permeability:kx=1.1×10−15ϕ2.5(1−ϕ)−0.33,

where *ϕ*_min⁡_ = 1 − *π*/4 [22]. The physiological range of porosity in ligaments is 0.32 ~ 0.42 [22]. The structure of an interosseous membrane is similar to that of ligaments. Therefore, the longitudinal permeability is approximately 1.0 × 10^−16^ m^2^, and the ratio of *k*_*x*_ to *k*_*y*_ (ratio = *k*_*x*_/*k*_*y*_) is approximately 10.

### 2.5. Boundary Conditions

The calculation domain is shown in Figure 1(b) and contains one whole and two half capillary clusters. The capillary wall is defined as the velocity-inlet boundary, and the inlet velocity is defined by UDF (8). In addition, because of the periodic geometric character, we define periodic boundary conditions as shown in Figure 1(b). The outward flow (at the bottom boundary) is fully developed, and the *x*-direction derivative of *u*_*x*_ is zero (∂*u*_*x*_/∂*x* = 0). The upstream flow is neglected, and the inlet velocity (the upper boundary) is defined to be zero. The calculation domain is a porous zone, and the viscous resistance is $1/{\overset{⃑}{k}}_{p}$.

When a mast cell is present in the interstitium, the mast cell region (the sphere in the center) is removed from the original calculation region. The sphere surface is presumed to be a wall; therefore, non-slip boundary conditions (*u*_*r*_ = 0, *u*_*θ*_ = 0) are used. The Brinkman boundary layer (*δ*) has a thickness of magnitude $\sqrt{{\overset{⃑}{k}}_{p}}$.

### 2.6. Computational Method

The CFD software package FLUENT (version 6.0) is used for the numerical simulation. The grid is generated using the GAMBIT software package. The model is laminar, the solver is segregated and steady, and the solving method is the SIMPLE scheme. The governing equations are solved by iterating. When the iteration is convergent (error of iterated results *e* < 0.001), the velocity field is obtained.

### 2.7. Physiological Parameters

Table 1 shows the physiological parameters used in the numerical simulation. 

## 3. Results

### 3.1. Flow Field without Interstitial Cell

**Figure 2:**
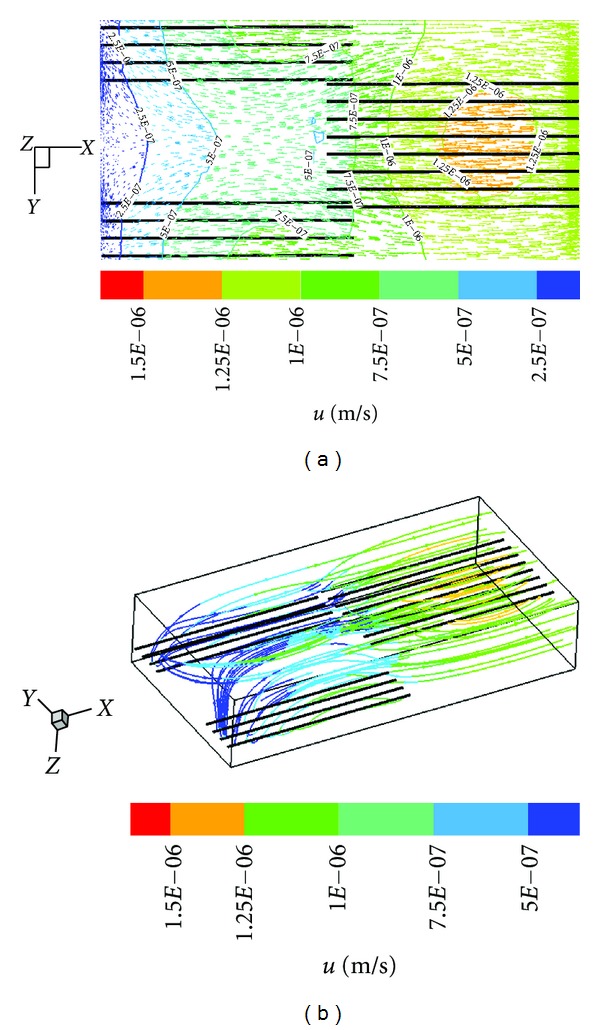
(a) Flow field in the *x*-*y* plane; (b) path lines from the capillaries.

Figure 2(a) is the flow field in the *x*-*y* plane (*z* = 0, display scale  : *y* = 1 : 2.5, *x* : *z* = 1 : 2.5). The thick black lines represent the capillaries, the arrows point in the direction of the velocity, the arrow length indicates the magnitude of the velocity, and the colored lines are contours of velocity. The interstitial fluid flows from the capillary to the interstitium on the upstream (left) side. Near the *x*-axis, the flow direction tends to become parallel to the capillaries. At the venule side, a small amount of the fluid is absorbed by capillaries while most fluid flows outward. In the first cluster, the inlet velocity is zero. However, the penetrating flux through the capillary wall is greater than the absorbing flux. Therefore, fluid flows out at the end of the first cluster. In the second cluster, because there are fluid flows out of the first cluster, the inlet velocity is no longer zero. Comparing the interstitial fluid velocity in the first cluster with that in the second cluster shows that the velocity in the second cluster is obviously greater. This difference occurs because the inlet velocity of the second cluster, which is generated from the first cluster of capillaries, accelerates the interstitial fluid. The outflow of the second cluster is greater than that of the first cluster. Therefore, the velocity in the third cluster will be even greater.  Figure 2(b) shows the path lines from the capillaries in the interstitial space (display scale  : *y* = 1 : 2.5, *x* : *z* = 1 : 2.5). The colored lines represent path lines, which are nearly parallel to the capillaries, and the maximum velocity is approximately 1.5 × 10^−6^ m/s (~2.5 U). 

### 3.2. Flow Field around an Interstitial Cell

**Figure 3:**
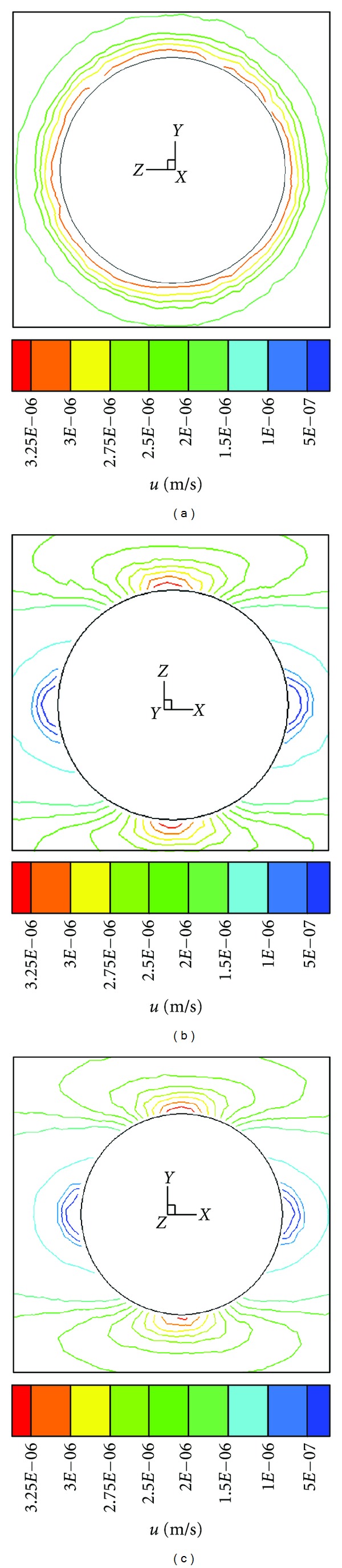
Streamlines of velocity around the cell. (a) *y*′-*z*′ plane; (b) *x*′-*z*′ plane; (c) *y*′-*z*′ plane.

Using this model, we also investigate the flow field with a cell in the interstitial space. The interstitial cell has little effect on the flow field except near the cell surface. The maximum velocity occurs at the cell surface (3.25 × 10^−6 ^m/s, 5 U) and is much greater than the velocity at other locations. The flow field near the cell is nearly symmetrical about the *x*′-axis.  Figure 3 displays the stream lines around the cell, with (a) showing the streamlines in the local *y*′-*z*′ plane (*x*′ = 0, represents the cross section), (b) showing the streamlines in the local *x*′-*z*′ plane (*y*′ = 0, represent the *r*-*θ* plane), and (c) showing the streamlines in the local *x*′-*y*′ plane (*z*′ = 0). Figures 3(b) and 3(c) are nearly identical. The direction of the velocity is along the *x*-axis, and the maximum velocity is at *x*′ = 0 mm.

### 3.3. Mechanical Environment of Interstitial Cell

**Figure 4:**
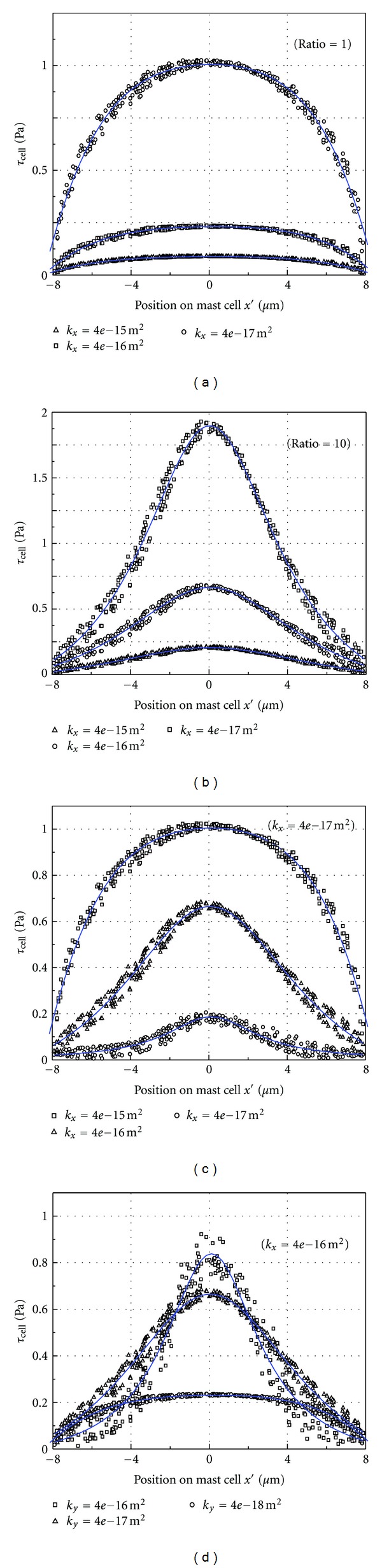
*τ*_cell_ distribution on the surface of the mast cell.

Interstitial fluid flow provides mechanical stimuli to the interstitial cell. The shear stress on the surface of the mast cell (*τ*_cell_) is calculated using (6). The shear stress is affected by the Darcy permeability *k*.  Figure 4 shows the *τ*_cell_ distribution on the cell surface for (a) an isotropic condition (ratio = 1) and (b) an anisotropic condition (ratio = 10). The results show that *τ*_cell_ has bilateral symmetry and that the maximum *τ*_cell_ (*τ*_cell, max⁡_) is at *x*′ = 0 mm for ratio ≥ 1 (ratio = 1 and ratio = 10). *τ*_cell_ increases as *k*_*x*_ decreases. As ratio increases, the *τ*_cell_ near *τ*_cell, max⁡_ changes significantly. The *τ*_cell_ distribution on the cell surface in Figure 4 is also shown for (c) fixed *k*_*y*_ (*k*_*y*_ = 4 × 10^−17^ m^2^) and (d) fixed *k*_*x*_ (*k*_*x*_ = 4 × 10^−16^ m^2^). The results show that *k*_*x*_ is the main factor affecting *τ*_cell_. When *k*_*y*_ is fixed, *τ*_cell_ increases as *k*_*x*_ decreases (Figure 4(c)). When *k*_*x*_ is fixed, as *k*_*y*_ decreases, the distribution of *τ*_cell_ becomes more uneven (Figure 4(d)). 

### 3.4. The Effect of Physiological Parameters on *τ*_cell, max⁡_

**Figure 5:**
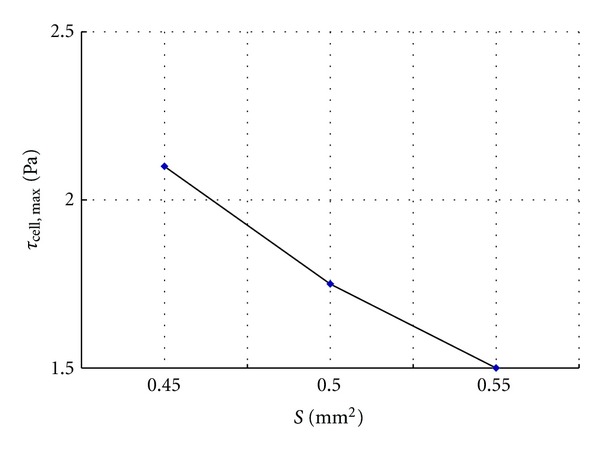
Variation of *τ*_cell, max⁡_ with cross-sectional area *S*.

The capillary density in the interstitium can affect the interstitial fluid flow and *τ*_cell_. With the cross-sectional area defined as *S* = *d*_*y*_∗*d*_*z*_, Figure 5 shows the relationship between *τ*_cell, max⁡_ and *S*. Obviously, *τ*_cell, max⁡_ decreases as *S* increases, and the relationship is nonlinear. For smaller *S*, larger changes occur in *τ*_cell, max⁡_.

**Figure 6:**
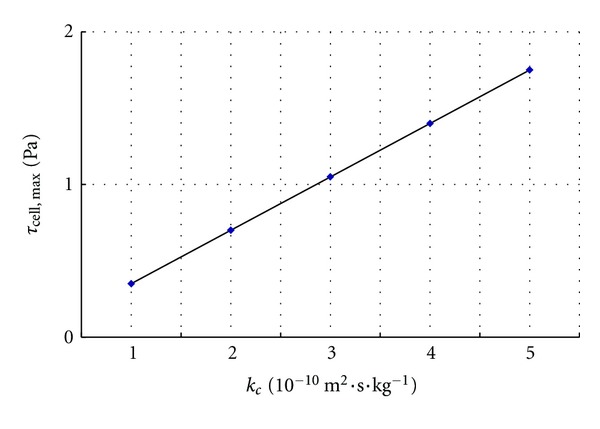
Variation of *τ*_cell, max⁡_ with *k*_*c*_.

The permeability coefficient of the capillary wall (*k*_*c*_) can also affect the interstitial fluid flow and *τ*_cell_. As shown in Figure 6, *τ*_cell, max⁡_ increases linearly with *k*_*c*_.

**Figure 7:**
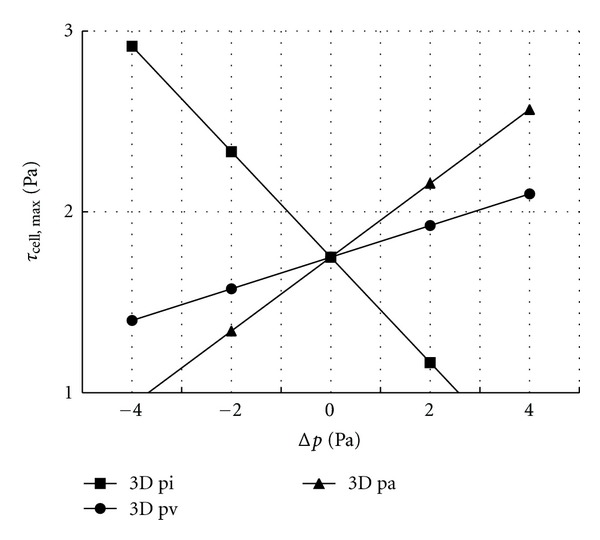
*τ*_cell, max⁡_ as *p*_*a*_, *p*_*v*_, and *p*_*i*_ change.

Both the intravascular capillary pressure (*p*_*c*_) and the interstitial hydrostatic pressure at the capillary wall (*p*_*i*_) can affect the permeability velocity of the capillary wall (7) and thus the interstitial fluid flow and *τ*_cell_.  Figure 7 shows how changes in *p*_*i*_, intravascular capillary pressure at the upstream end (*p*_*a*_), and intravascular capillary pressure at the downstream end (*p*_*v*_) affect *τ*_cell, max⁡_. The abscissa is the change in *p*_*a*_, *p*_*v*_, and *p*_*i*_ compared to the standard values *p*_*a*0_, *p*_*v*0_, and *p*_*i*0_, respectively; that is, Δ*p*_∗_ = *p*_∗_ − *p*_∗0_.*τ*_cell, max⁡_ increases linearly with increasing *p*_*a*_ and *p*_*v*_, and decreases linearly with increasing *p*_*i*_. The effect of *p*_*i*_ is the greatest, whereas the effect of *p*_*v*_ is the least.

Based on the numerical results, we can make the following observations. Capillaries in a parallel array can induce parallel interstitial fluid flow. Interstitial fluid flow can induce shear stress on the cell surface. The ratio determines the distribution of the shear stress, and *k*_*x*_ greatly affects the maximum shear stress. The shear stress can be increased by decreasing the cross-sectional area, which corresponds to increasing the capillary density. The shear stress can also be increased by increasing capillary permeability, increasing intravascular capillary pressure and decreasing interstitial pressure.

## 4. Discussion

### 4.1. Directional Interstitial Flow May Explain Some Experimental Observations

The velocity field shows that the direction of the interstitial fluid flow is parallel to the orientation of the capillaries. The first cluster of capillaries can generate an interstitial fluid flow with a velocity of approximately 0.75 × 10^-6 ^m/s. This flow then enters the space surrounding the second cluster of capillaries and accelerates to 1.25 × 10^-6 ^m/s. The maximum velocity in the space surrounding the second cluster of capillaries is 1.5 × 10^-6 ^m/s. If the fluid is not absorbed by the lymphatic system, it will flow downstream and be accelerated by the downstream capillaries. In the past, it was accepted that most of the seepage from the arteriole side of a capillary is absorbed at the venule side, and the surplus is immediately absorbed by lymphatic vessels. In reality, capillaries are not always near lymphatic vessels, and the amount of fluid that seeps from capillaries is always greater than the amount of fluid absorbed by capillaries. Therefore, the unabsorbed fluid will travel some distance or even a long distance before being reabsorbed by blood or lymphatic vessels. It is possible that the long interstitial tracks observed in a previous experiment are in fact the interstitial fluid flow [16]. The tracer (Gd-DTPA) injected into the acupoint travels with the interstitial fluid flow. Therefore, the observance of flow along a meridian that differs from blood and lymph flow may be interstitial fluid flow [15]. 

### 4.2. A Staggered Distribution of Capillaries Results in a Uniform Interstitial Fluid Velocity

The distribution of the capillaries can influence the velocity distribution of the interstitial fluid flow. Figure 2(a) shows this influence. In the first cluster, the capillaries are located farther from the *x*-axis, and thus the interstitial fluid velocity farther from the *x*-axis increases more quickly than the velocity near the *x*-axis. In contrast, the capillaries in the second cluster are located near the *x*-axis, and this interstitial fluid velocity near the *x*-axis increases faster than the flow farther from the *x*-axis. With this staggered distribution of capillaries, the increases in velocity alternate, and the velocity tends to be uniform, which is beneficial for substance exchange. Interstitial fluid flow will affect the bioactivities of cells. When tissues are poorly vascularized, such as ligaments and tendons, interstitial fluid flow is more important for metabolism than it is for well-vascularized tissues.

### 4.3. The Shear Stress Induced by Interstitial Fluid Flow Will Affect Cell Bioactivities

The numerical simulation shows that the flow has a velocity of magnitude 10^-6 ^m/s and that the flow induces several Pa shear stresses on the mast cell surface. Many studies showed that subtle fluid mechanical environment played an important role in the ability of cells to proliferate, differentiate, form functional structures, and release chemical mediators [26, 27]. Recent studies showed that interstitial flow influences the immune microenvironment in cancer [28]. The immune microenvironment of the tumor consists of multiple cell types, cytokines, and stromal components that can further attract immune cells and guide their fate. Civelek et al. presented the first direct evidence that SMCs with a contractile phenotype will indeed contract when exposed to 2D fluid shear stress [29]. Studies suggest that both laminar shear stress (2D) and interstitial flow (3D) can induce SMC contraction [30]. These studies provide a different perspective regarding the mechanism for myogenic control of blood flow that regulates flow distribution in response to blood pressure changes. Wang found intracellular Ca^2+^ increases in mast cells and mediators release immediately after mechanical stimulation [31]. Further research showed that the mechanosensitive Ca^2+^ channel TRPV_2_ may be involved in this process [32]. 

The mechanism for how interstitial flow affects cell bioactivities is still unknown. It was once generally believed that the shear stress induced by the flow was the main factor. Further studies suggest that cell membrane-related receptors, ion channels, cell surface glycocalyx, integrins, and signaling messengers are all involved in this process. A recent paper shows that the fluid shear stress on cells is rather small and that the solid stress induced by interstitial flow through cell surface glycocalyx is much higher [33]. The solid stress more likely plays a major role in regulating cell function and behavior. However, it is difficult to evaluate solid stress because doing so requires detailed knowledge of the glycocalyx microstructure and extracellular matrix properties. In this paper, we do not focus on the complex mechanism of membrane ion channel activation and solid stress induced by interstitial flow through the cell surface glycocalyx. Instead, we only compare the shear stresses on the mast cell surface in different conditions.

### 4.4. Physiological Parameters Variation Has Effect on *τ*_cell_

The longitudinal permeability and the ratio determined the distribution of *τ*_cell_. From (9), we know that *k*_*x*_ and *k*_*y*_ are functions of the porosity *ϕ*. Therefore, *ϕ* affects *τ*_cell_. Actually, the smaller *ϕ* is, the greater effect on *τ*_cell, max⁡_ is. At normal physiological values, *ϕ* has much smaller effects than when the values are outside of the normal physiological range [19]. Increasing intravascular capillary pressure or capillary permeability can increase *τ*_cell_. Therefore, blood microcirculation can affect the living conditions of interstitial cells, and changing the blood supply is an effective method for adjusting the circulation of interstitial fluid. Decreasing the interstitial pressure can also increase *τ*_cell_, and doing so has a greater impact than changing the intravascular capillary pressure. 

### 4.5. TCM Treatments and Acupuncture Effect

With traditional Chinese treatments such as massage and cupping, the interstitial pressure can be changed by applying periodic or negative pressure on the body surface [34]. The above results show that interstitial pressure is a main factor affecting interstitial flow and *τ*_cell_, Therefore, there may be a correlation between these treatments and interstitial flow.

Acupuncture is a therapeutic treatment in which a needle is inserted into specific parts (acupoints). When the needle is twirled, lifted and thrusted, the winding of collagen on the acupuncture needle changes the interstitial microenvironment [35, 36], which produces mechanical stimulation. The stimulation causes degranulation in local mast cells and the release of biological mediators such as histamine, substance P, and leukotriene C_4_, and so forth [14]. These mediators can further activate mast cells and excite nerve endings, which may lead to “De-qi” (a local sensation of heaviness, numbness, soreness, or paresthesia, which is believed to be an important aspect of acupuncture treatment). Moreover, these mediators have a powerful effect in increasing capillary permeability and increasing interstitial flow [24]. The increased flow not only increases the *τ*_cell_ to activate local mast cells but also transports biological mediators secreted by mast cells to activate other mast cells along the flow path [2, 37]. In summary, the meridian phenomena and the “De-qi” sensations along the meridian during acupuncture may be closely related to the interstitial flow.

## Figures and Tables

**Table 1:** Physiological parameter values of the model.

Parameter	Value
Viscosity of interstitial fluid *μ*/(kg·m^−1^·s^−1^)	3.5 × 10^−3^ [22]
Permeability coefficient of capillary wall *k*_*c*_/(m^2^·s·kg^−1^)	5 × 10^−10^ [23]
Plasma colloid osmotic pressure *π*_*c*_/mmHg	28 [24]
Interstitial colloid osmotic pressure at the capillary wall *π*_*i*_/mmHg	8 [24]
Density of interstitial fluid *ρ*/(kg·m^−3^)	1000
Length of capillary *L*/*μ*m	2000
Diameter of capillary *D*/*μ*m	8 [19]
Distance between adjacent capillaries *d*/*μ*m	48 [19]
Interstitial hydrostatic pressure at the capillary wall *p*_*i*_/mmHg	−5 [24]
Intravascular capillary pressure at the upstream end *p*_*a*_/mmHg	25 [24]
Intravascular capillary pressure at the downstream end *p*_*v*_/mmHg	10 [24]
Width of capillary group *W* (*μ*m)	392
Length of the calculation domain *d*_*x*_ (*μ*m)	3600
Width of the calculation domain *d*_*y*_ (*μ*m)	784
Height of the calculation domain *d*_*z*_ (*μ*m)	632
